# *Schizostachyum dakrongense* (Poaceae, Bambusoideae), a new species from Dakrong Nature Reserve, Vietnam

**DOI:** 10.3897/phytokeys.138.39623

**Published:** 2020-01-10

**Authors:** Zhuo-Yu Cai, Yi-Hua Tong, Tien-Chinh Vu, Jing-Bo Ni, Nian-He Xia

**Affiliations:** 1 Key Laboratory of Plant Resources Conservation and Sustainable Utilization/Guangdong Provincial Key Laboratory of Digital Botanical Garden, South China Botanical Garden, Chinese Academy of Sciences, 510650, Guangzhou, China South China Botanical Garden, Chinese Academy of Sciences Guangzhou China; 2 University of Chinese Academy of Sciences, 100049, Beijing, China University of Chinese Academy of Sciences Beijing China; 3 Southeast Biodiversity Center, Xishuangbanna Tropical Botanical Garden, Chinese Academy of Sciences, China Vietnam National Museum of Nature, Vietnam Academy of Science and Technology Hanoi Vietnam; 4 Vietnam National Museum of Nature, Vietnam Academy of Science and Technology, Hanoi, Vietnam Graduate University of Science and Technology Hanoi Vietnam; 5 Graduate University of Science and Technology, Vietnam Academy of Science and Technology, Hanoi, Vietnam Xishuangbanna Tropical Botanical Garden, Chinese Academy of Sciences Xishuangbanna China

**Keywords:** Asia, Melocanninae, morphology, taxonomy, woody bamboos

## Abstract

*Schizostachyum dakrongense* is a new species of woody bamboo from Dakrong Nature Reserve, Quang Tri Province, central Vietnam. It is closely related to *S. hainanense* but differs by its pseudospikelets having terminal rachilla segments with ciliate margin and 6 bracts; culm sheath with the base of the outer margin having a slight projection below its point of attachment at the node, as well as sheath blades usually less than half as long as the culm sheath proper; and leaf blades pale-puberulent and sparsely pilosulous on the abaxial surface. The new species is described and illustrated here.

## Introduction

*Schizostachyum* Nees, which was established by Nees von Esenbeck in 1829, is a genus of subtribe Melocanninae (Poaceae, Bambusoideae) (Nees von Esenbeck 1829; BPG 2012). It is widely distributed in tropical and subtropical southeastern Asia (Wong 1995; Widjaja 1997; Ohrnberger 1999; Xia and Stapleton 2006). This genus is closely related to several later genera: *Teinostachyum* Munro, *Neohouzeaua* A. Camus, *Dendrochloa* Parkinson, *Leptocanna* Chia & H.L. Fung, *Cephalostachyum* Munro, and *Pseudostachyum* Munro so that there are different opinions about generic delimitation (Munro 1868; McClure 1935; Holttum 1946; Dransfield 1983; Xia 1993; Yang et al. 2007; Vorontsova et al. 2016). One opinion, suggested by Xia, is that *Teinostachyum*, *Neohouzeaua*, *Dendrochloa*, and *Leptocanna* should be combined with *Schizostachyum*, and both *Cephalostachyum* and *Pseudostachyum* should be recognized as a separate genus (Xia 1993). Molecular evidence has supported this suggestion (Yang et al 2007; Zhou et al 2017).

The first species of *Schizostachyum* in Vietnam was discovered by Balansa, who reported *S. zollingeri* Steud. (Balansa 1890). E.G. Camus also recorded this species in his monograph (Camus 1913). Several years later, E.G. Camus and A. Camus found another species of *Schizostachyum* in Vietnam, namely *S. aciculare* Gamble (Camus and Camus 1923). In 1942, McClure recorded two further species, *S. pseudolima* McClure and *S. hainanense* Merr. ex McClure (McClure 1942). Besides the above-mentioned four species, Pham recorded another seven species of *Schizostachyum* bamboo in Vietnam (Pham 2000). In the Bamboos of Vietnam, Nguyen increased this number to sixteen, with many undescribed species (Nguyen 2006). But after systematically researching Vietnam *Schizostachyum*, Tran (2011) thought that six species previously determined as *Schizostachyum* were in fact misidentifications. In his revision, he followed Xia’s concept and recognized that there were fifteen species of *Schizostachyum* in Vietnam, including several undescribed ones. Up to now, he has published 4 new species of Vietnam *Schizostachyum*, namely *S. ninhthuanense* N.H. Xia, V.T. Tran & H.N. Nguyen, *S. yalyense* N.H. Xia, V.T. Tran & H.N. Nguyen, *S. nghianum* N.H. Xia & V.T. Tran, and *S. langbianense* V.T. Tran, N.H. Xia & H.N. Nguyen (Tran et al. 2010, 2013, 2016).

Dakrong Nature Reserve is located in Quang Tri province, central Vietnam. The main terrain of the reserve includes low ranges that are part of the Annamite Mountains. With a tropical monsoon climate, the average annual temperature of this area is 22−24 °C and the average annual precipitation is 2500−3000 mm. There are large areas of lowland forest in the reserve. These forests are located in a zone of overlap between the tropical Indo-Pacific/Sunda and subtropical/temperate China floristic regions. Consequently, this area shows high species richness and diversity (Trai et al. 1999). About 1053 species of plants are recorded for the 40,253 ha area (CRES 2005).

During a field survey in Dakrong Nature Reserve in November 2018, we collected a flowering bamboo which appeared similar to *S. hainanense*. But after further study, we confirmed that it is an undescribed species characterized by a ciliate margin in the terminal rachilla segment of the pseudospikelet and the base of the culm sheath’s outer margin developing a slight projection below the point of attachment at the node.

## Materials and methods

Material from this new species was collected from the type locality. Flowers were dissected under an Olympus-SZX16 microscope and photomicrographs microphotos were taken with a Qimaging MicroPublisher 3.3 RTV instrument. Morphological comparisons were based on characters recorded in the relevant literature including protologues, as well as a study of type specimens. The type specimens, photos and living plants were used for describing this new species.

## Taxonomic treatment

### Schizostachyum dakrongense

Taxon classificationPlantaePoalesPoaceae

N.H.Xia, Z.Y.Cai, Y.H.Tong & T.C.Vu sp. nov.

29519B14-66D4-5C47-BA35-6E596E0E011C

urn:lsid:ipni.org:names:77204209-1

Figures 1 , 2

#### Type.

Vietnam. Quang Tri Province: Dakrong Nature Reserve, 16°37'16.80"N, 106°52'1.80"E, alt. ca. 200m, 15 Nov. 2018, *N.H. Xia et al* BVN20181114 (holotype: IBSC!; isotype: VNMN!).

#### Diagnosis.

This new species resembles *Schizostachyum hainanense* in having culm sheaths with an asymmetrically concave apex and well-developed oral setae. However, *S. dakrongense* differs from *S. hainanense* in its pseudospikelets having a terminal rachilla segment with ciliate margin, and 6 bracts; the base of the culm sheath outer margin with a slight projection below its point of attachment; culm sheath blades that are usually less than half as long as the sheath proper; pale-puberulent and sparsely pilosulous leaf blade abaxial surfaces.

**Figure 1.**
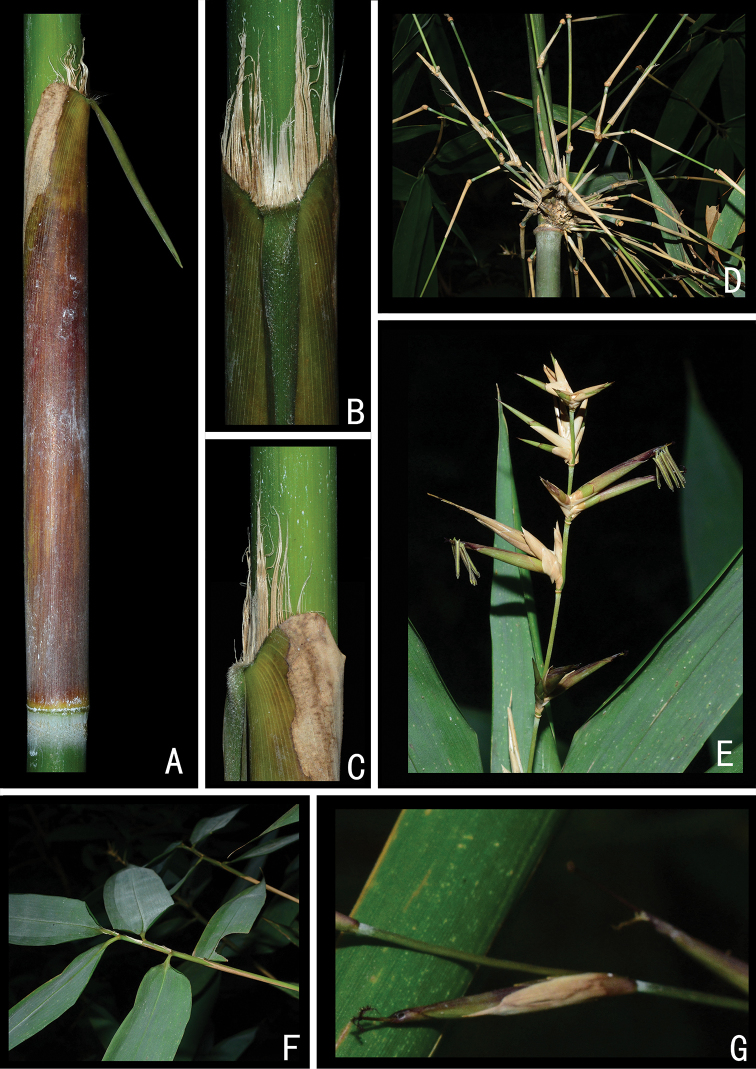
Morphological character of *S. dakrongense* sp. nov. **A** Culm sheath **B, C** apex of culm sheath **D** branches **E** pseudospikelets and anthers **F** foliage blades **G** pseudospikelets and stigmas.

#### Description.

Culms erect, apex pendulous, 6–8 m tall, ca. 1.5 cm in diameter; internodes ca. 40 cm long, slightly siliceous and scabrous, with sparse white hairs, sometimes with white waxy powder, with an especially conspicuous white-powdery and brown-yellow setaceous zone ca. 1 cm wide just below each node; walls 1–2 mm thick. Culm sheaths up to 23 cm long, dark brown, covered with white powder and appressed brown hairs, margins sparsely ciliate, apex asymmetric, concave (ca. 9 mm deep), base of outer margin with a slight projection below point of attachment; auricles absent; oral setae well-developed, up to 20 mm or longer; ligule up to ca. 1 mm long, fringes of margin ca. 10 mm long; culm sheath blade narrowly lanceolate, reflexed, entire, usually less than half as long as culm sheath, adaxial side covered with dense white and brown hairs, especially at the base. Branches numerous and subequal, 30–50 cm long. Foliage leaves complements with 6–10 leaves; leaf blades oblong-lanceolate or linear-lanceolate, 10–21 cm long, 1.3–3.7 cm wide, adaxial surface slightly scabrous, abaxial surface pale-puberulent and sparsely pilosulous; sheaths 3–8 cm long, glabrous; auricles absent; oral setae well developed, pale, 10–15 mm long; ligule up to ca. 0.5 mm long, margin fimbriate. Pseudospikelets with 1 floret, clustered on leafy flowering branches, fusiform, ca. 20 mm long; prophylls ca. 2.5 mm long, ovate-lanceolate or triangular, apex acute or emarginate, abaxial surface glabrous or hairy; bracts (5−)6, ovate or ovate-lanceolate, apex emarginate, obtuse to acute or mucronate, abaxial surface glabrous or hairy, margin ciliate or not, the lowest bract without buds in its axils, the top two each with a bud in their axils, the middle ones with a bud in their axil or not; glumes absent; rachilla ca. 1 mm long, terminal segment enlarged, disciform, margin ciliate; lemma ca. 12 mm long, ovate-lanceolate, involute, apex acuminate mucronate, many-veined, margins ciliate or not; palea ca. 15 mm, strongly involute, upper portion indurate, lower portion soft, apex mucronate; lodicules absent; filaments white, ca. 13 mm long, free, anthers ca. 6 mm long, brownish yellow; ovary ovoid, glabrous, style ca. 15 mm long, stigmas 3, purple, ca. 1.5 mm long, plumose. Fruit unknown.

**Figure 2.**
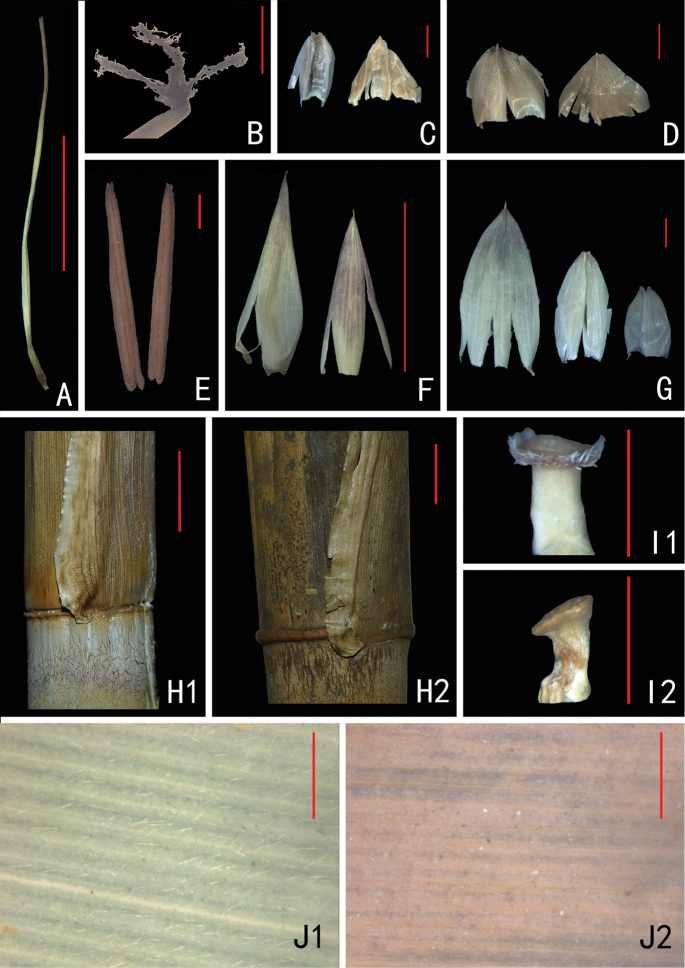
Morphological character of *S. dakrongense* sp. nov. and comparisons between *S. dakrongense* and *S. hainanense*. **A** Ovary and style **B** stigmas **C** prophylls **D, G** bracts **E** stamens **F** Palea (left) and Lemma (right) **H1** base of culm sheath outer margin of *S. dakrongense***H2** base of culm sheath outer margin of *S. hainanense***I1** rachilla of *S. dakrongense* with ciliate margin **I2** rachilla of *S. hainanense* with glabrous margin **J1** Leaf abaxial surface of *S. dakrongense* pale-puberulent and sparsely pilosulous **J2** leaf abaxial surface of *S. hainanense* glabrous. Scale bars: 1 cm (**A, F, H1, H2**); 1 mm (**B–E, G, I1, I2, J1, J2**).

**Table 1.** Morphological comparisons of *S. dakrongense* with *S. hainanense*.

Characters	*S. dakrongense*	*S. hainanense*
Culm	6−8 m tall, ca. 1.5 cm in diameter	8−20(−30) m tall, ca. 4 cm in diameter
Internodes	ca. 40 cm long	75 cm long or more
sheath blade	usually less than half as long as sheath	usually more than half as long as sheath
Base of culm sheath outer margin	With a slight projection below point of attachment	With a conspicuous semi-circular projection below point of attachment
Leaf abaxial surface	pale-puberulent and sparsely pilosulous	glabrous
Bracts	(5−)6	3−4
Rachilla terminal segment	margin ciliate	glabrous

#### Etymology.

The species epithet “dakrongense” refers to the type locality: Dakrong Nature Reserve, Quang Tri Province, Vietnam.

#### Phenology.

New shoots are found from summer to autumn.

#### Distribution and habit.

According to our investigations and the available data, *S. dakrongense* is only distributed in Dakrong Nature Reserve. It commonly occurs in evergreen forest at an elevation of ca. 200 m, together with *Eurycoma longifolia* Jack, Ficus hirta var. roxburghii King, *Archidendron occultatum* (Gagnep.) I.C. Nielsen, and a species of *Ochna*.

#### Conservation status.

The species is found in a protected area, so its environment appears to be relatively stable. It is locally common. However, the area of distribution is less than 400 km^2^. According to International Union for Conservation of Nature (IUCN) Red List categories and criteria, this species should be treated as Endangered (EN) (IUCN 2012).

## Supplementary Material

XML Treatment for Schizostachyum dakrongense
